# Prognostic Significance of Negative Lymph Node Long Axis in Esophageal Cancer: Results From the Randomized Controlled UK MRC OE02 Trial

**DOI:** 10.1097/SLA.0000000000005214

**Published:** 2023-01-10

**Authors:** Maximilian Kloft, Jessica E. Ruisch, Gayatri Raghuram, Jake Emmerson, Matthew Nankivell, David Cunningham, William H. Allum, Ruth E. Langley, Heike I. Grabsch

**Affiliations:** * Department of Pathology, GROW School for Oncology and Reproduction, Maastricht University Medical Center+, Maastricht, Netherlands;; † Division of Pathology and Data Analytics, Leeds Institute of Medical Research at St James’s, University of Leeds, Leeds, UK;; ‡ Department of Pathology, Nottingham University Hospitals, Nottingham, UK;; § Leeds Institute of Clinical Trials Research, University of Leeds, Leeds, UK;; ¶ MRC Clinical Trials Unit at UCL, University College London, London, UK;; || Gastrointestinal and Lymphoma Unit, Royal Marsden Hospital, London, UK; and; ** Department of Surgery, Royal Marsden Hospital, London, UK.

**Keywords:** esophageal cancer, immune system, lymph nodes, neoadjuvant chemotherapy, survival

## Abstract

To analyze the relationship between negative lymph node (LNneg) size as a possible surrogate marker of the host antitumor immune response and overall survival (OS) in esophageal cancer (EC) patients.

**Background:**

Lymph node (LN) status is a well-established prognostic factor in EC patients. An increased number of LNnegs is related to better survival in EC. Follicular hyperplasia in LNneg is associated with better survival in cancer-bearing mice and might explain increased LN size.

**Methods:**

The long axis of 304 LNnegs was measured in hematoxylin-eosin stained sections from resection specimens of 367 OE02 trial patients (188 treated with surgery alone (S), 179 with neoadjuvant chemotherapy plus surgery (C+S)) as a surrogate of LN size. The relationship between LNneg size, LNneg microarchitecture, clinicopathological variables, and OS was analyzed.

**Results:**

Large LNneg size was related to lower pN category (*P* = 0.01) and lower frequency of lymphatic invasion (*P* = 0.02) in S patients only. Irrespective of treatment, (y)pN0 patients with large LNneg had the best OS. (y)pN1 patients had the poorest OS irrespective of LNneg size (*P* < 0.001). Large LNneg contained less lymphocytes (*P* = 0.02) and had a higher germinal centers/lymphocyte ratio (*P* = 0.05).

**Conclusions:**

This is the first study to investigate LNneg size in EC patients randomized to neoadjuvant chemotherapy followed by surgery or surgery alone. Our pilot study suggests that LNneg size is a surrogate marker of the host antitumor immune response and a potentially clinically useful new prognostic biomarker for (y)pN0 EC patients. Future studies need to confirm our results and explore underlying biological mechanisms.

Esophageal cancer (EC) is the sixth most common cause of cancer-related death worldwide with 604,100 new cases and 544,076 deaths in 2020.^1,2^ Standard of care for patients with resectable (cT2N0 or higher) EC is currently neoadjuvant chemo(radio)therapy followed by surgical resection.^3,4^ The UK Medical Research Council (MRC) OE02 trial was the first phase III trial showing the superiority of neoadjuvant chemotherapy followed by surgery over surgery alone, changing clinical practice.^4^

When determining the prognosis for EC patients, the N status (number of lymph nodes [LNs] with metastatic disease) is one of the most important prognostic factors.^5,6^ An increasing number of regional LN metastases (‘positive’ lymph nodes [LNpos]) has been associated with a poorer prognosis in EC patients and patients with other cancer types.^7–9^ There is also evidence to suggest that the LN ratio (number of LNpos/total number LNs) has prognostic value in EC patients.^10^ Furthermore, it has been proposed recently that an increased number of LNs without metastasis (‘negative’ lymph nodes [LNneg]) is associated with improved overall survival (OS) in patients with esophageal squamous cell cancer (SCC).^11^ Although most studies in the past focused on evaluating the prognostic value of the number of LNpos or the LN ratio,^12^ there are only few studies investigating the prognostic value of the LNpos size in EC patients with SCC, most based on radiological imaging in patients with metastatic disease.^13,14^ EC patients with larger LNpos seem to have a poorer survival than those with smaller LNpos.^15–17^

It has been suggested that regional, primary tumor draining LNs have a key role in the host antitumor immune response and that increasing LNneg size might be related to a better prognosis.^18^ Indeed, a recent study in colorectal cancer patients suggested that the presence of large LNneg is related to a longer progression free survival.^19,20^ A study in Dukes B rectal cancer patients suggested that large LNneg are related to increased recurrence free survival and increased histologic antitumor response.^21^ Furthermore, follicular hyperplasia in LNneg has been associated with better survival in mice with cancer and might explain increased LN size.^22^ These findings were confirmed in colon cancer patients indicating that an increased number of large LNneg is related to an increase in primary tumor infiltrating lymphocytes.^23^

A recent computed tomography imaging-based study suggested that chemotherapy effects LNpos size in EC patients and the extent of downsizing may be related to patients long-term prognosis.^24^ However, the current literature is still controversial regarding the effect of chemotherapy on LNneg, and it is not clear whether chemotherapy increases or decreases LNneg size.^25,26^ Mice studies suggest that the size of LNneg changes depending on primary tumor regression or progression.^22^ The histopathological characterization of LNneg in a small group of EC patients treated by surgery alone found patterns of increased immunosuppression in LNnegs of pathological (p)N1 patients compared to pN0 patients.^27^

To the best of our knowledge there has been no study investigating the relationship between histologically measured LNneg size and survival in EC patients treated with either surgery alone (S patients) or neoadjuvant chemotherapy followed by surgery (C+S patients).

We hypothesized that EC patients with large LNneg at the time of resection have a better survival regardless of treatment modality.

The aim of the present pilot study was to measure the long axis of LNnegs as a surrogate of LNneg size in the resection specimens of 367 EC patients from the OE02 trial and investigate the relationship between LN size, clinicopathological variables including treatment and patient OS.

## Methods

**Figure 1:**
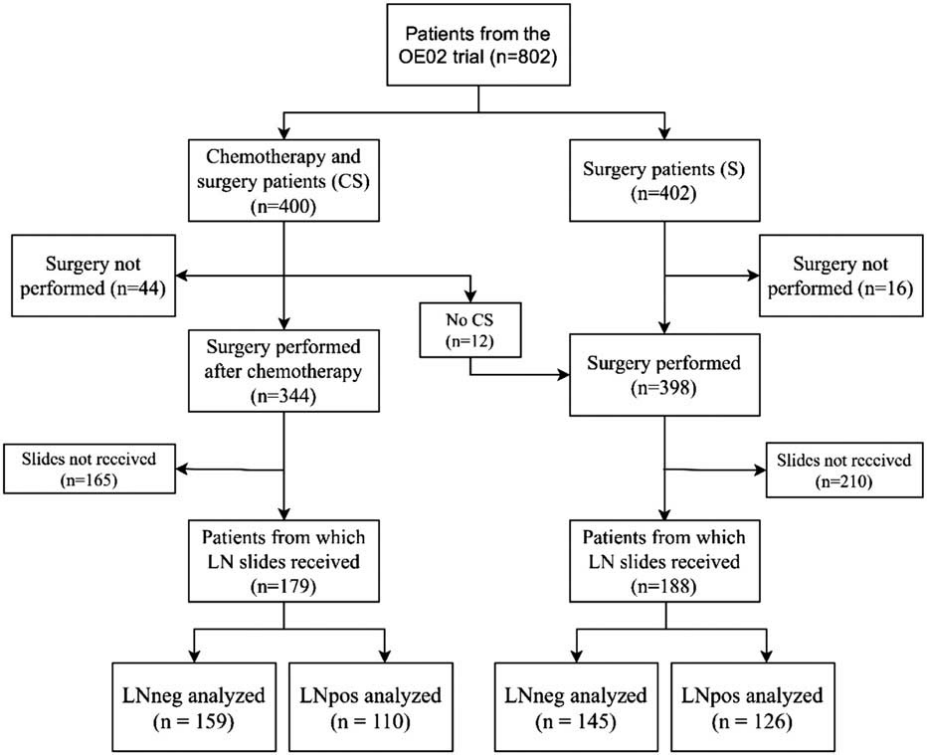
Flow diagram showing the number of patients originally randomized in the OE02 trial to either neoadjuvant chemotherapy and surgery or surgery alone and for whom lymph node sizes could be determined from virtual histopathological slides. LNneg, negative lymph nodes; LNpos, positive lymph nodes.

In the UK MRC OE02 trial, 802 patients with histologically or cytologically confirmed, locally advanced resectable EC were randomized to treatment by surgery alone (S patients) or neoadjuvant chemotherapy consisting of 2 cycles of 5-fluorouracil and cisplatin followed by surgery (C+S patients) between 1992 and 1998.^3,4^ For inclusion and exclusion criteria and details about patient allocation and precise treatment schedules see publication of the clinical results.^3^ In total, 344 C+S patients and 398 S patients proceeded to surgical resection in the trial. Hematoxylin/eosin slides and paraffin blocks from the resection specimen were collected retrospectively. Slides with LNs were available from 179 C+S patients and 188 S patients for analyses (Fig. 1). This represents 48% of the OE02 trial patients who had a resection. Cases were not preselected, all available resected LNs were used for analyses. The REMARK checklist was used for reporting of the methods and the results^28^ (see Supplemental Digital Content Table 1, http://links.lww.com/SLA/D443).

**Table 1:** Clinicopathological Characteristics for Chemotherapy Plus Surgery patients and Surgery Alone Patients Stratified by LNneg Size (Cutoff Point = Median 7.41 mm)

	All Patients With LNneg		Chemo+Surgery					Surgery Alone				
	LNneg Size <7.41 mm		LNneg Size ≥7.41 mm		LNneg Size <7.41 mm			LNneg Size >7.41 mm		LNneg Size ≥7.41 mm	
Characteristics	n	%	n	%	n	%	*P*	n	%	n	%	*P*
Sex												
Male	222	73	59	75	65	81	0.36	56	77	42	58	0.05
Female	82	27	20	25	15	19		17	23	30	42	
Age at diagnosis (median)	62.3		63.3		59.6		0.12	64.1		59.2		0.08
Location of primary tumor												
Lower	199	64	55	69	56	70	0.54	45	61	43	60	0.79
Middle	70	23	18	23	15	19		18	25	19	26	
Upper	35	13	6	8	9	11		10	14	10	14	
Histology of primary tumor												
AC	213	70	57	72	18	23	0.89	52	23	21	29	0.41
SCC	73	24	17	22	57	73		17	71	47	65	
Other	18	6	5	6	5	4		4	6	4	6	
(y)pT												
T0^*^	8	3	5	6	3	4	0.12	0	0	0	0	0.75
T1	19	6	3	4	9	10		5723	
T2	30	10	6	8	11	14		5	7	8	11	
T3	238	78	61	78	57	71		60	82	60	85	
T4	8	3	4	4	0	0		3	4	1	1	
(y)pN												
N0	93	31	26	33	27	34	0.82	13	18	27	37	0.01
N1	211	69	53	67	53	66		60	82	45	63	
Grade of differentiation												
Moderate/well	154	51	40	51	47	59	0.53	32	44	35	48	0.34
Poor	134	44	32	41	27	34		40	56	35	48	
Unknown	16	5	7	8	6	7		1	1	2	4	
Lymphatic invasion												
No	199	64	58	73	59	74	0.96	33	45	49	67	0.02
Yes	105	36	21	27	21	26		39	55	24	33	
Blood vessel invasion												
No	166	55	73	92	74	92	0.98	58	79	61	85	0.37
Yes	36	45	6	6	6	8		15	21	9	15	
Resection margin status												
Negative	199	65	50	64	59	74	0.15	46	63	44	61	0.85
Positive	80	26	24	30	15	18		20	27	21	29	
Unknown	45	9	5	6	6	8		7	10	7	10	
Tumor regression grade primary tumor											
TRG 1–3	39	25	21	27	18	23	0.67					
TRG 4–5	135	75	58	73	62	77						

Location primary tumor refers to lower, middle and upper thirds of the esophagus.

* T0: No residual tumor in the specimen.

AC, adenocarcinoma; SCC, squamous cell carcinoma; pT, depth of invasion according to UICC TNM classification 6th ed.;pN, lymph node status accroding to UICC TNM classification 6th ed.

Clinicopathological data of this subset of OE02 trial patients such as histological tumor type, grade of tumor differentiation, tumor regression grade according to Mandard classification,^29^ depth of invasion ((y)pT category) and LN status ((y)pN category) were established during central histopathology review or extracted from the original pathology reports (tumorsize, tumorlocation, numberof LNs, resection margin status) and classified according to Union for International Cancer Control (UICC) Tumour Node Metastasis (TNM) classification 6th edition.^30^ Clinical outcome data were extracted from the UK MRC OE02 clinical trial database. The study was approved by the South East Research Ethics Committee, London, UK, REC reference: 07/H1102/111.

The clinicopathological data from our study cohort were compared to all OE02 trial patients who had a resection to confirm the representativeness of our subset.

### Measurement of LN Long Axis

Hematoxylin/eosin stained slides with tumor free regional LNs (LNneg) were available from 159 C+S patients and 145 S patients. Regional LNs with tumor metastasis (LNpos) were available from 110 C+S patients and 126 S patients (Fig. 1).

Slides were scanned at 40 × magnification using an Aperio XT Scanner and reviewed via a web interface using ImageScope (Aperio ImageScope v11.2.0.780, Leica, Milton Keynes, UK).

A LN was defined as any size of lymphoid tissue with a clearly identifiable capsule or as an aggregate of lymphoid tissue without capsule measuring >3 mm in long axis according to the LN definition of UICC TNM classification 5th ed.^31^ An irregular border was also regarded as the outline of the LN. Two aggregates of lymphoid tissue were considered as one LN if they were less than 1 mm apart or were present in the same piece of fatty tissue on the same slide (see Supplemental Digital Content Figure 1, http://links.lww.com/SLA/D443). LNnegs with signs of tumor regression such as fibrosis or mucin lakes without viable tumor cells which therefore must have been tumor positive before treatment (n = 12), were excluded from analysis.

The LN border was manually annotated along the outer border in ImageScope using a pen tool and touch screen (Wacom Cintiq 15x pl-550 15 LCD tablet, Krefeld, Germany). Length measurement lines were placed manually using the ‘ruler tool’ and measurements in millimeter were provided by the software. The investigator drawing the LN outlines and placing the measurement lines was blinded to any clinicopathological parameters including patient treatment details. The LN status (positive vs negative), outlines and long axis lines were quality controlled by a second independent investigator.

### Assessment of Negative LN Microarchitecture

To better understand the relationship between LNneg size and survival in EC patients without LN metastasis, we decided to explore the LN microarchitecture features in more detail in these patients. We quantified the LN specific microarchitectural features using point counting with random systematic sampling, a well-established technique for morphometric object quantification.^32^ We used 250 measurement points ±5% tolerance for each LN (see Supplemental Digital Content Figure 2, http://links.lww.com/SLA/D443). Each measurement point was manually reviewed and the tissue type at the tip of the arrow was categorized as lymphocytes (outside of a germinal center [GC]), GC, histiocytes, vessels, other tissue (fat, connective tissue) or noninformative (artifacts, arrow outside LN capsule etc.) at 5× magnification (see Supplemental Digital Content Figure 2, http://links.lww.com/SLA/D443 and Supplemental Digital Content Figure 3, http://links.lww.com/SLA/D443). For each LNneg, the percentage of area (%area) covered with a particular microarchitecture feature was calculated as follows:

**Figure 2:**
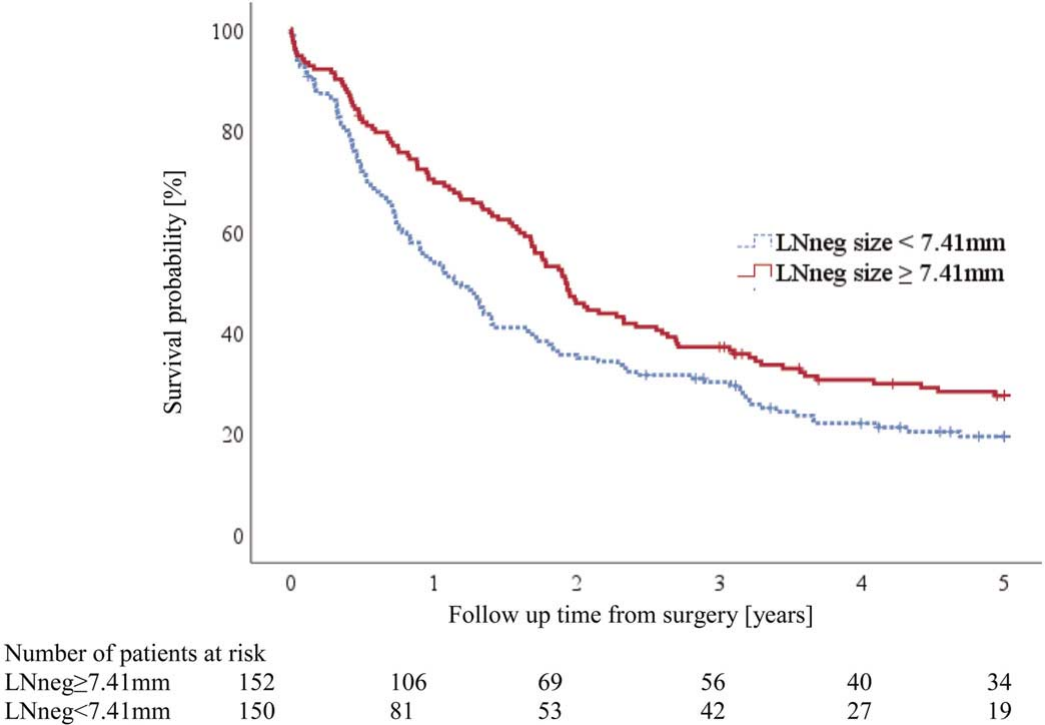
Five-year overall survival (OS) stratified by size of the negative lymph node (LNneg). LNneg size was dichotomized at the median (7.41 mm). The Kaplan-Meier plot shows that esophageal cancer patients with larger LNneg survive significantly longer than those with smaller LNneg. Hazard Ratio (HR): 0.73, 95% Confidence Interval (CI): 0.56–0.94, *P* = 0.017. Five-year OS 28% vs 19%.

**Figure 3:**
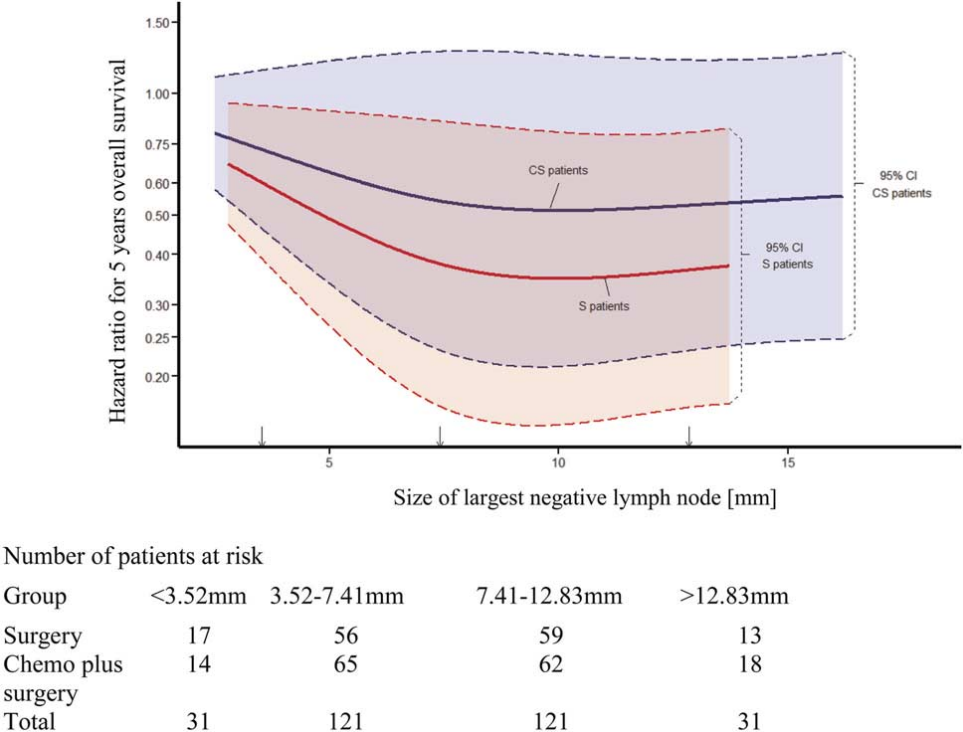
Restricted cubic spline plot illustrating the relationship between risk of death (hazard ratio) and negative lymph node size separately for S patients (red line) and C+S patients (blue line). The arrows mark the knots identified with the restricted cubic spline approach at 3.52 mm, 7.41 mm, and 12.83 mm. The dashed lines and shaded areas highlight that the confidence intervals for the 2 treatment groups are largely overlapping. Note that the function used to create the curves does not utilize the lower and upper 5% of data points as extreme values can have a large distorting effect on the curve leading to potential misinterpretation. This results in the surgery alone curve being shorter than the chemotherapy plus surgery curve. In both treatment arms, the risk of death decreases with increasing LNneg size up to a lowest point around the second knot at 7.41 mm. The effect of the LNneg size on the risk of death seems to be larger in the S patients. However, due to the overlapping confidence intervals and probably related to relatively small sample size, this difference is not significant.


$$\begin{array}{ll} \%\text{area}= & \frac{100}{\text{total}\;\text{number}\;\text{of}\;\text{informative}\;\text{points}}\\ \times \text{number}\;\text{of}\;\text{points}\;\text{with}\;\text{a}\;\text{particular}\;\text{feature} \end{array}$$

Additionally, ratios of particular microarchitectural features were calculated per LNneg: %area of GC/%area of lymphocytes, %area of histiocytes/%area of lymphocytes, %area of GC/%area of histiocytes.

### Statistical Analyses

Statistical analyses were performed using SPSS statistics software (version 25, IBM, Hampshire, England) and R (version 3.5.3).^33^ The length of the LN long axis was used as a surrogate of LN size. The largest LNneg and the largest LNpos per patient were used for statistical analyses. All available samples were included. In case of missing data of LNneg or LNpos size, patients were excluded from the respective analyses.

As we were also interested in a potential effect of neoadjuvant chemotherapy on LN size, LNpos size and LNneg size were compared within treatment arms and between treatment arms. Similarly, the relationship between LN size, histological tumor type (adenocarcinoma vs squamous cell carcinoma [SCC]), predominant grade of tumor differentiation according to WHO classification, depth of invasion ((y)pT), LN status ((y)pN), and resection margin status was investigated for LNneg and LNpos separately per treatment arm. TNM staging was calculated according to TNM classification 6th ed. as this was the TNM classification valid at the time of the original trial reporting. The Kruskal-Wallis test was used to determine if statistically significant differences were present between 2 or more groups.

As there were no data published which cutoff to use when analyzing the histological LN size, we initially used the radiological LN size cutoff of 10 mm for categorizing LNs as being large (≥10 mm) versus small (<10 mm).

To assess the relationship of LNneg size and survival, we fitted a restricted cubic spline as described by Gauthier et al^34^ to the data for each treatment arm, using the R package, Hmisc.^35^ Splines model the relationship between size of largest LNneg and hazard ratio of 5-year OS with individual piecewise curves drawn between a selected number of knot points. The number of knots is chosen based on the number of data points available. Knot points are selected based on percentiles of LNneg size to ensure that each curve is informed from an even amount of data. The ‘restricted’ element comes from restricting the individual curves so that they must meet at the knot points to form one smooth curve across LNneg size. From this curve it can be assessed whether there is an increased or decreased hazard ratio of 5-year OS across different values of LNneg size.

For this study, a 3-knot placement for LNneg size was selected to maximize the information used to create the spline curves. These knot points would be utilized as cut-points to categorize LNneg size within the multivariate and univariate model if the relationship was found to be nonlinear. The plot resulting from this analysis allowed us to visually assess whether there was a sufficiently large difference in the relationship between LNneg size and survival between treatment arms which would make it necessary to add an interaction term to the multivariate survival model. We chose a per protocol analysis to accurately access the effects of the different treatments.

The primary endpoint in this study was 5-year OS. Five-year OS was calculated from the date of surgery to the date of death or last follow up using the Kaplan-Meier method. Survival differences were assessed using log-rank statistics. As there was no survival difference between C+S and S patients in our cohort (see Result section), survival analyses were performed for the whole cohort initially.

Multivariate survival analysis was performed using a Cox proportional hazard model including the covariates final treatment (surgery alone vs neoadjuvant chemotherapy followed by surgery), (y)pT category, (y)pN status, and LNneg size. For additional analysis, the variable of the cubic spline approach and interaction treatment-LNneg size were included as covariates in the Cox proportional hazard model. Proportional hazards were tested using the Kaplan-Meier plots.

In addition, we explored whether a potential survival benefit of the LNneg size could be related to LN status ((y)pN0 vs (y)pN1). For this analysis we combined LNneg size dichotomized at the median (LNneg size <7.41 mm or ≥7.41 mm) and (y)pN status and compared OS of patients with (y)pN0+ LNneg<median vs (y)pN0+LNneg≥median vs (y)pN1 + LNneg<median vs (y)pN1+LNneg≥median using the Kaplan-Meier method. The median LNneg size of all patients (including (y)pN0 and (y)pN1 patients) was 7.41 mm, which was used as a cutoff point.

In the (y)pN0 patient subcohort, LN microarchitectural features (%area and ratios) were compared between patients with large LNneg (≥median LNneg size of N0 patients [8.13 mm]) and small LNneg (<8.13 mm) using Kruskal-Wallis test. A cutoff point of 8.13 mm was used as this was the median LNneg size of (y)pN0 patients. Boxplots were created to visualize the difference of the respective LN microarchitectural feature between groups of LNneg size.

*P* values of <0.05 were considered significant. For an overview of the performed analyses, see the REMARK profile, Supplemental Digital Content Table 2, http://links.lww.com/SLA/D443. For an overview of used cutoffs see Supplemental Digital Content Table 3, http://links.lww.com/SLA/D443.

**Table 2:** Univariate and Multivariate Overall Survival Analysis in the Study Cohort

	Univariate		Multivariate	
		HR (95% CI)	*P*	HR (95%CI)	*P*
Sex				
*Male versus female*	1.23 (0.94–1.63)	0.14		
Age at diagnosis	1.03 (1.0101.04)	**<0.001**		
Treatment				
*Chemotherapy plus surgery versus surgery alone*	1.14 (0.9–1.44)	0.28	0.98 (0.48–2.25)	0.96
Location primary tumor				
*Middle versus lower*	1.2 (0.89–1.62)	0.24		
*Upper versus lower*	0.67 (0.43–1.05)	0.08		
Histology primary tumor				
*AC versus SCC*	0.9 (0.69–1.19)	0.46		
*Other versus SCC*	0.84 (0.42–1.17)	0.62		
(y)pT status				
*T3/T4 versus T0-T1/T2*	1.5 (1.22–1.84)	**<0.001**	1.87 (1.29–2.87)	**0.002**
(y)pN status				
*N1 versus N0*	2.01 (1.5–2.68)	**<0.001**	1.56 (1.16–2.15)	**0.006**
Grade of differentiation				
*Poor versus moderate/well*	1.28 (0.98–1.67)	**0.07**		
Lymphatic invasion				
*Positive versus negative*	1.82 (1.38–2.4)	**<0.001**		
Blood vessel invasion				
*Positive versus negative*	1.96 (1.33–2.88)	**0.001**		
Resection margin status				
*Positive versus negative*	2.01 (1.56–2.59)	**<0.001**		
Tumor regression grade primary tumor				
*TRG 4/5 versus TRG 1/2/3*	1.45 (0.99–2.11)	0.06		
LNneg size				
*LNneg ≥7.41 mm versus LNneg <7.41 mm*	0.73 (0.57–0.95)	**0.019**		
Restricted cubic splines of LNneg size				
*Spline group 2 versus group 1*			0.79 (0.45–1.62)	0.46
*Spline group 3 versus group 1*			0.67 (0.38–1.37)	0.22
*Spline group 4 versus group 1*			0.93 (0.48–2.3)	0.87
Treatment interaction of LNneg size splines				
Treatment*spline group 2			1.18 (0.48–2.78)	0.71
Treatment*spline group 3			0.95 (0.36–2.13)	0.9
Treatment*spline group 4			0.74 (0.21–2.1)	0.6

Spline groups refer to cut-point defined groups of the restricted cubic spline approach.

* Group 1: <3.52 mm, group 2: 3.52–7.41 mm, group 3: 7.41 mm–12.83 mm, group 4: >12.83 mm.

AC, adenocarcinoma; SCC, squamous cell carcinoma; pT, depth of invasion according to UICC TNM classification 6th ed.;pN, lymph node status accroding to UICC TNM classification 6th ed.

## Results

The clinicopathological parameters such as age, sex, (y)pT and (y)pN stage, and OS of patients included in the current study were similar to those of all OE02 trial patients who had a resection (see Supplemental Digital Content Table 4, http://links.lww.com/SLA/D443 and Supplemental Digital Content Table 5, http://links.lww.com/SLA/D443, Supplemental Digital Content Figure 4, http://links.lww.com/SLA/D443). Therefore, patients included in our study cohort were considered representative of the OE02 trial population who had a resection.

**Figure 4:**
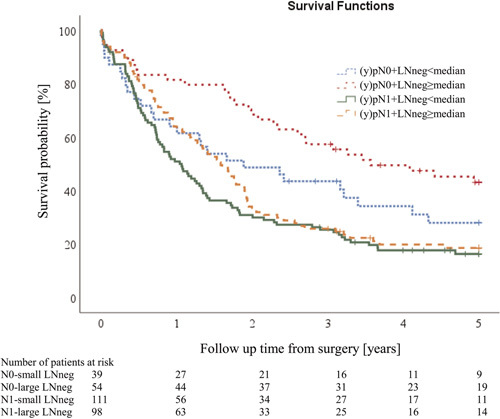
Five-year overall survival (OS) stratified by combined groups of (y)pN status and LNneg size. The Kaplan-Meier plot shows that the survival of EC patients differs significantly depending on the stratified group (*P* < 0.001). N0+LNneg<median: HR: 1.64. 95%CI: 0.97–2.76;*P* = 0.062; N1+LNneg<median: HR: 2.46, 95% C11.62–3.72, *P* < 0.001; N1+LNneg>median: HR: 2.07, 95% CI 1.36–3.17, *P* = 0.001 (reference group: N0+LNneg≥median). Five-year OS: N0-small LNneg: 26.9%, N0-large LNneg: 42.4%, N1-small LNneg: 16.5%, N1-large LNneg: 18.6%.

The median (range) age of patients (n = 304) included in the current study was 62.5 years (30–83.1 years). Median (range) follow up time from surgery was 16.9months (0.06–158.1 months). Median (range) number of resected LNs per patient was 10 (1–67).

Three hundred patients (81.7%) had died at the end of the study period. For a summary of clinicopathological data at the time of randomization and from the resection specimen stratified by LNneg size see Table 1, and Supplemental Digital Content Table 6, http://links.lww.com/SLA/D443 for LNpos.

### LN Size and Relationship With Clinicopathological Parameters

This pilot study included 367 patients in total, 179 C+S patients and 188 S patients (Fig. 1). Of these 367 patients, 176 (48%) had LNpos and LNneg available for measurement, 63 (17%) had only LNpos available and 128 (35%) had only LNneg available. In total, we measured 2058 LNs, of which 1041 were from C+S patients and 1017 were from S patients.

The largest LNneg size was similar between C+S patients and S patients (median [range] C+S: 7.53 mm [1.54–20.43 mm] vs S: 7.35 mm [1.38–24.49 mm], *P* = 0.5).

In S patients, a larger LNneg size was related to a lower number of LNpos (*P* = 0.018), lower pN status (*P* = 0.01), and lower frequency of lymphatic invasion (*P* = 0.02, Table 1). Largest LNneg size was not related to primary tumor location, histological tumor type (SCC vs adenocarcinoma), grade of tumor differentiation or blood vessel invasion (all *P* values > 0.05).

In C+S patients, largest LNneg size was not related to any of the clinicopathological characteristics (all *P* values > 0.05, Table 1).

The size of the largest LNpos differed significantly between C+S patients and S patients (median [range] C+S: 8.7 mm [2.43–25.56 mm] vs S: 10.97 mm [2.24–29.91 mm], *P* = 0.003). Largest LNpos size was not related to any of the clinicopathological characteristics neither in S patients nor in C+S patients (all *P* values >0.05, Supplemental Digital Content Table 6, http://links.lww.com/SLA/D443).

### Clinicopathological Parameters and Relationship With 5-year OS

Significant prognostic factors in univariate survival analysis were age at diagnosis (*P* < 0.001), (y)pT category (*P* = 0.001), (y)pN status (*P* < 0.001), grade of primary tumor differentiation (*P* = 0.01) and lymphatic invasion (*P* < 0.001) (Table 2).

### Negative LN Size and Relationship With 5-year OS

Using the radiologically used LN size cutoff of 10 mm, there was no survival difference between patient with LNneg size ≥10 mm and LNneg size <10 mm (Hazard Ratio (HR): 1.21, 95% Confidence Interval (CI): 0.91–1.62; *P* = 0.19) (see Supplemental Digital Content Figure 5, http://links.lww.com/SLA/D443).

**Figure 5:**
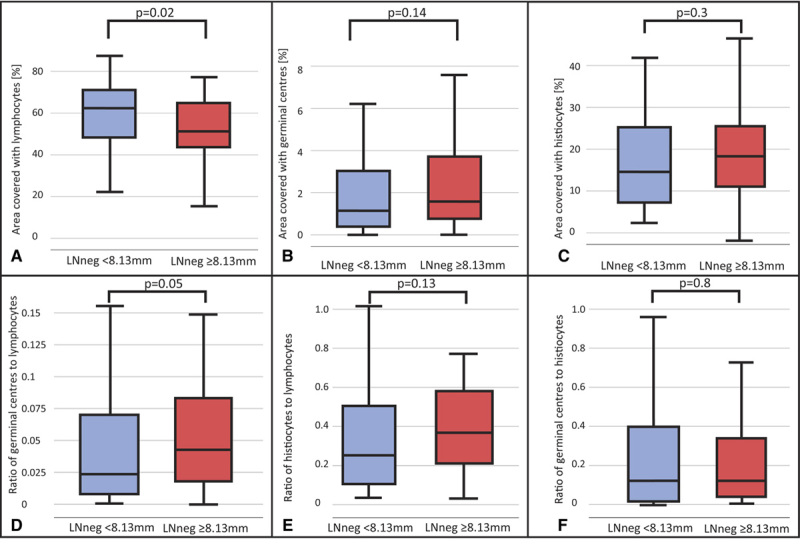
A–F, Boxplots showing the difference in microarchitecture between large and small LNneg in the NO subgroup of the OE02 cohort (n = 93). Panels A, B, and C showing percentage of area (%area) covered with lymphocytes, germinal centers, and histiocytes, respectively. Panels D, E, and F showing ratios of germinal centers to lymphocytes, histiocytes to lymphocytes, and ratio of germinal centers to histiocytes, respectively. Box represents 25th to 75th percentile, line in the box indicates the median, whiskers indicating lower and upper quartiles.

Using the restricted cubic spline approach with 3 internal knots (see Methods), we modelled the relationship between risk of death and maximum LNneg size per treatment arm. Figure 3 shows the spline curves for the 2 treatment arms including the respective confidence intervals. The curve shows a steadily decreasing risk of death with increasing LNneg size in both treatment arms approximately up to the median LNneg size of all patients (7.41 mm). For illustration, a Kaplan Meier graph of the whole patient cohort was plotted to compare OS between patients with LNneg size stratified at the median (Fig. 2). This illustrates the increased OS probability for patients with large LNneg (HR: 0.73; 95% CI: 0.56–0.94, *P* = 0.017).

For LNneg size greater than the median, the spline curves (Fig. 3) begin to level off indicating no change in risk of death by increasing LNneg size before possibly slightly raising after LNneg size of 13 mm. There is a suggestion from this restricted cubic spline approach, that the relationship between LNneg size and OS may be non-linear.

Although we appreciated that both treatments showed somehow similar trends in their splines with respect to survival and despite confidence intervals were largely overlapping, the distance between the curves of S and C+S patients was felt to be sufficiently big enough to justify including an interaction term for treatment within the multivariate model. The 3 internal knots were used as cut-points to categorize the LNneg size variable into 3 groups within the multivariate survival models to reflect the difference across the knot groups.

### Multivariate Analysis

Multivariate analysis with the categorial covariates treatment, (y)pT status, (y)pN status, LNneg size groups by spline cut-points, and treatment interaction of LNneg groups by spline cut-points confirmed (y)pN status (HR: 1.56 [1.16–2.15], *P* = 0.006) and (y)pT status (HR: 1.87 [1.29–2.87], *P* = 0.002) as independent prognostic variables. None of the other variables including LNneg size were significant in multivariate analysis (Table 2). There was no significant treatment interaction.

### Exploratory OS Analysis Combining (y)pN Status and Negative LN Size

We explored whether the LNneg size parameter provides additional information to the LN status and categorized patients according to their LN status and LNneg size into 4 groups: (y)pN0+LNneg<median; (y)pN0+LNneg≥median; (y)pN1 + LNneg<median; (y)pN1 +LNneg≥median. Univariate survival analysis showed a significant survival difference between groups (*P* < 0.001, Fig. 4). (y)pN0 patients with large LNneg had the best OS, followed by (y)pN0 patients with small LNneg, whereas (y)pN1 patients had the poorest survival irrespective of LNneg size. Interestingly, the survival of the (y)pN1 patients seems to be better during the first 2 years in the presence of large LNneg, whereas the survival of (y)pN1 patients seems to be no longer related to LNneg size after 2 years.

### Negative LN Microarchitecture of (y)pN0 Patients

To find out whether microarchitectural changes of the largest LNneg in the (y)pN0 patients might be related to the better survival of some of the ypN0 patients, we analyzed the LN microarchitecture features of (y)pN0 patients and their relationship with LNneg size.

In (y)pN0 patients with small LNneg (LNneg size <8.13 mm), the median percentage of LN area (%area) with lymphocytes was 61.1% (range: 22.6%–84.6%) compared to 50.9% (range: 10.3%–75.3%) in (y)pN0 patients with large LNneg (LNneg size ≥8.13 mm), *P* = 0.02 (Fig. 5A–C). The %area of GCs was higher in (y)pN0 patients with large LNneg, although not reaching statistical significance (median [range] 1.6% [0%–15.4%] vs 1.2% [0%–8.7%], *P* = 0.14). The %area containing histiocytes did not differ between patients with small and large LNneg (13.2% [2.3%–63.1%] vs 20.1% [0%–73%]; *P* = 0.2). The GCs/lymphocytes ratio was higher in patients with large LNneg with borderline significance (0.03 [0–0.3] vs 0.02 [0–0.14]; *P* = 0.05), suggesting that large LNnegs may have increased formation of GC structures compared to small LNnegs (Fig. 5D–F).

## Discussion

Research aiming to identify new potential prognostic or predictive biomarker has mainly focused on the primary tumor or on the number of positive LNs, although the potential prognostic impact of features that characterize LNs without metastasis has not been investigated in detail in patients with EC. Earlier studies in colorectal cancer suggested that the size of the LNneg could be an indicator of increased host antitumor immune response and could be related to a survival advantage.^20,21,23^

The current study aimed to explore the relationship between the LN size and prognosis in EC patients from the UK MRC OE02 trial treated with surgery alone (S patients) or neoadjuvant chemotherapy followed by surgery (C+S patients). We used the length of the long axis of a LN as surrogate of LN size. Overall, the LN sizes in our study were similar to that found by others.^36^ We found that patients with large LNneg in the resected specimen survived significantly longer than patients with small LNneg. Thus, this is the first study to suggest a potential prognostic role of LNneg size in EC patients irrespective of treatment modality or histological tumor type. Our EC results are concordant with previous findings reported in colorectal and gastric cancer.^36–38^

LN status (pN) is known to be one of the most important prognostic factors in EC patients, more important than primary tumor regression grade as we showed in a previous study in the OE02 trial patients.^6^ Although LNneg size was not an independent prognostic marker in multivariate analysis, our exploratory analysis combining the LNneg size with the LN status revealed that (y)pN0 EC patients with large LNneg have a better survival than (y)pN0 EC patients with small LNneg. This could potentially suggest that the LNneg size might be a clinically useful marker to identify (y)pN0 patients who may benefit from further adjuvant treatment.

Size changes in regional LNneg in pancreatic cancer and colorectal cancer patients have been related to an increased host antitumor immune response resulting in follicular hyperplasia with the formation of GCs, proliferation of lymphocytes in the medullary or paracortical area and/or sinus histiocytosis due to incoming tumor derived antigens.^39,40^ It has been shown that secondary follicles not only become hyperplastic but may also develop GCs and enlarge their underlying dendritic network possibly explaining the overall changes in LN size during immune response activation.^41–43^ Our pilot study of the LN microarchitecture found that large LNnegs in (y)pN0 patients have significantly less lymphocytes located outside of GCs and a higher ratio of GCs to lymphocytes confirming studies showing immune response leading to LN size changes.^44,45^ This could possibly provide an explanation for the improved OS of (y)pN0 patients with large LNneg.

A retrospective study in colorectal cancer suggested that a higher number of LNneg might be related to an increased lymphocyte infiltration in the primary tumor and better survival.^46^ Unfortunately, LN size measurements were not included in this study, so results cannot be directly compared with our findings.^46^ In the current study in EC patients, we found a relationship between large regional LNneg and lower number of LNpos. However, related to the relatively small number of patients in subgroups, we are unable to distinguish between a survival benefit related to enlarged LNneg (eg, due to augmented immune activation in regional tumor draining LN) and a survival benefit due to overall lower metastatic burden.

To the best of our knowledge, the effect of chemotherapy on the histological size of regional LNneg has not been investigated in EC resection specimens. As the OE02 trial had a surgery alone arm and a chemotherapy plus surgery arm, we were able to assess whether neoadjuvant chemotherapy induces a change in the size of the LNneg and whether the prognostic value of the LNneg size was different between treatment arms. To our surprise, neither LNneg size nor prognostic value of large LNneg size were different between treatment arms. We had expected that due to the chemotherapy induced immunogenic cell death, LNneg size would increase.^19^ Indeed, LNneg size was found to be increased in rectal cancer patients treated with chemoradiotherapy, compared to patients treated with surgery alone.^47^ In contrast, Schröder et al^48^ did not find a difference in LNneg size when comparing LNneg size in a small series of EC SCC patients treated with either surgery alone or chemoradiotherapy. It should be emphasized that the aforementioned 2 studies combined chemotherapy with radiotherapy which makes direct comparison to our findings difficult.

The current study has some limitations. We used retrospectively collected material from a trial which recruited EC patients between 1992 and 1998. The OE02 trial protocol did not include specific guidance for the surgeons, regarding the type of LN dissections nor for the pathologist, on handling the resection specimen. We therefore had to assume that it was common practice to cut through the center of the LN at the time of specimen cut up by pathologists. Reassuringly, the LN sizes measured in the current study are comparable to those reported by others in more recent studies.^36^ LN sizes can also increase in case of an infection, clinical data to this effect is unavailable but presence of an infection could have influenced our results. The total number of resected LNs found per patient would be considered relatively low compared to current standards. This could be related to the LN dissection by the surgeon and/or pathologist, as the type of lymphadenectomy is unknown but also to the unavailability of the slides to us after such a long time of material storage. We chose to analyze the largest LN size when patients had multiple LNs assuming that large LNs are more likely resected and found in the resected specimen by the pathologist than small ones. A larger number of LNs for all patients might have allowed a more detailed analysis of the effect of number of large LNs or combination of large LNneg with LNpos sizes. Unfortunately, data on the LNneg location were not available consequently we could not relate this to the tumor location. Future analysis in larger groups of patients including LNpos is needed.

In summary, this pilot study using resection material from EC patients randomized to the OE02 trial provides first insights into the potential importance of assessing LNneg size to predict prognosis in EC patients and in particular to identify EC patients with (y)pN0 status with a poorer prognosis who might benefit from additional postsurgical therapy. Our results require validation in an independent cohort and further studies to better understand the underlying biological mechanism of increased LNneg size in some patients. Based on our results, we hypothesize that large regional LNneg size identified at the time of diagnosis might be a potential clinically useful marker for the identification of highly immunogenic EC. Thus, there is a clinical need to improve radiological staging of LNs and in particular recognition of LNneg. This might be achieved in the first instance by combining pathology-based LN size studies with matched radiological imaging studies at the time of surgery to translate histological findings into patient management decisions in the future and personalize risk-stratification in EC patients.

## Supplementary Material

## References

[1] Bray F Ferlay J Soerjomataram I. Global cancer statistics 2018: GLOBOCAN estimates of incidence and mortality worldwide for 36 cancers in 185 countries. CA Cancer J Clin. 2018;68:394–424. 30207593 10.3322/caac.21492

[2] Ferlay J Ervik M Lam F. Global Cancer Observatory: Cancer Today. International Agency for Research on Cancer; 2020.

[3] Medical Research Council Oesophageal Cancer Working Group. Surgical resection with or without preoperative chemotherapy in oesophageal cancer: a randomised controlled trial. Lancet. 2002;359:1727–1733. 12049861 10.1016/S0140-6736(02)08651-8

[4] Allum WH Stenning SP Bancewicz J. Long-term results of a randomized trial of surgery with or without preoperative chemotherapy in esophageal cancer. J Clin Oncol. 2009;27:5062–5067. 19770374 10.1200/JCO.2009.22.2083

[5] Mariette C Piessen G Briez N. The number of metastatic lymph nodes and the ratio between metastatic and examined lymph nodes are independent prognostic factors in esophageal cancer regardless of neoadjuvant chemoradiation or lymphadenectomy extent. Ann Surg. 2008;247:365–371. 18216546 10.1097/SLA.0b013e31815aaadf

[6] Davarzani N Hutchins GGA West NP. Prognostic value of pathological lymph node status and primary tumour regression grading following neoadjuvant chemotherapy - results from the MRC OE02 oesophageal cancer trial. Histopathology. 2018;72:1180–1188. 29465751 10.1111/his.13491 PMC5969086

[7] Ho AS Kim S Tighiouart M. Metastatic lymphnode burden and survival in oral cavity cancer. J Clin Oncol. 2017;35:3601–3609. 28880746 10.1200/JCO.2016.71.1176 PMC5791830

[8] Dubecz A Kern M Solymosi N. Predictors of lymph node metastasis in surgically resected T1 esophageal cancer. Ann Thorac Surg. 2015;99:1879–1885. discussion 86. 25929888 10.1016/j.athoracsur.2015.02.112

[9] Rice TW Apperson-Hansen C DiPaola LM. Worldwide Esophageal Cancer Collaboration: clinical staging data. Dis Esophagus. 2016;29:707–714. 27731549 10.1111/dote.12493 PMC5591441

[10] Zhang H Wang W Diao D. Ratio of metastatic to examined lymph nodes, a helpful staging system and independent prognostic factor of esophagogastric junction cancer. PLoS one. 2013;8:e73238. 23977381 10.1371/journal.pone.0073238 PMC3747090

[11] Zhu Z Chen H Yu W. Number of negative lymph nodes is associated with survival in thoracic esophageal squamous cell carcinoma patients undergoing three-field lymphadenectomy. Ann Surg Oncol. 2014;21:2857–2863. 24740827 10.1245/s10434-014-3665-y

[12] Wang N Jia Y Wang J. Prognostic significance of lymph node ratio in esophageal cancer. Tumour Biol. 2015;36:2335–2341. 25412956 10.1007/s13277-014-2840-x

[13] Dhar DK Tachibana M Kinukawa N. The prognostic significance of lymph node size in patients with squamous esophageal cancer. Ann Surg Oncol. 2002;9:1010–1016. 12464595 10.1007/BF02574521

[14] Chi Y-K Chen Y Li X-T. Prognostic significance of the size and number of lymph nodes on pre and post neoadjuvant chemotherapy CT in patients with pN0 esophageal squamous cell carcinoma: a 5-year follow-up study. Oncotarget. 2017;8:61662–61673. 28977894 10.18632/oncotarget.18665 PMC5617454

[15] Sugawara K Yamashita H Uemura Y. Preoperative lymph node status on computed tomography influences the survival of pT1b, T2 and T3 esophageal squamous cell carcinoma. Surg Today. 2019;49:378–386. 30467719 10.1007/s00595-018-1741-9

[16] Zhao Z Zhang Y Wang X. The prognostic significance of metastatic nodal size in non-surgical patients with esophageal squamous cell carcinoma. Front Oncol. 2020;10:523. 32373526 10.3389/fonc.2020.00523 PMC7176819

[17] Mine S Watanabe M Imamura Y. Clinical significance of the pretherapeutic nodal size in patients undergoing neo-adjuvant treatment followed by esophagectomy for esophageal squamous cell carcinoma. World J Surg. 2017;41:184–190. 27468743 10.1007/s00268-016-3675-y

[18] Rotman J Koster BD Jordanova ES. Unlocking the therapeutic potential of primary tumor-draining lymph nodes. Cancer Immunol Immunother. 2019;68:1681–1688. 30944963 10.1007/s00262-019-02330-y PMC6805797

[19] Okada K Sadahiro S Chan L. The number of natural killer cells in the largest diameter lymph nodes is associated with the number of retrieved lymph nodes and lymph node size, and is an independent prognostic factor in patients with stage II colon cancer. Oncology. 2018;95:288–296. 30138925 10.1159/000491019 PMC6262683

[20] Märkl B Rößle J Arnholdt HM. The clinical significance of lymph node size in colon cancer. Mod Pathol. 2012;25:1413–1422. 22684222 10.1038/modpathol.2012.92

[21] Murphy J Pocard M Jass JR. Number and size of lymph nodes recovered from dukes B rectal cancers: correlation with prognosis and histologic antitumor immune response. Dis Colon Rectum. 2007;50:1526–1534. 17828403 10.1007/s10350-007-9024-3

[22] Grundmann E Vollmer E. DMH-induced experimental carcinogenesis in the rat intestine [chapter 2]. In: Reaction Patterns of the Lymph Node. Springer Berlin Heidelberg; 1991. 2–6.

[23] Markl B Wieberneit J Kretsinger H. Number of intratumoral T lymphocytes is associated with lymph node size, lymph node harvest, and outcome innode-negative coloncancer. Am J Clin Pathol. 2016;145:826–836. 27329640 10.1093/ajcp/aqw074

[24] Urakawa S Makino T Yamasaki M. Lymph node response to neoadjuvant chemotherapy as an independent prognostic factor in metastatic esophageal cancer. Ann Surg. 2021;273:1141–1149. 31274656 10.1097/SLA.0000000000003445

[25] Hayashi Y Nishida T Tsujii M. Lymph node enlargement after definitive chemoradiotherapy for clinical stage I esophageal squamous cell carcinoma. BMC Cancer. 2014;14:706. 25253238 10.1186/1471-2407-14-706 PMC4193190

[26] Whitson BA Runge SJ Groth SS. False-positive mediastinal lymph node activity on positron emission tomographic scan after adjuvant treatment of gynecologic malignancies. J Thorac Cardiovasc Surg. 2007;133:1385–1386. 17467472 10.1016/j.jtcvs.2007.01.017

[27] Otto B Koenig AM Tolstonog GV. Molecular changes in pre-metastatic lymph nodes of esophageal cancer patients. PLoS One. 2014;9:e102552. 25048826 10.1371/journal.pone.0102552 PMC4105535

[28] McShane LM Altman DG Sauerbrei W. Reporting recommendations for tumor marker prognostic studies (REMARK). J Natl Cancer Inst. 2005;97:1180–1184. 16106022 10.1093/jnci/dji237

[29] Mandard AM Dalibard F Mandard JC. Pathologic assessment of tumor regression after preoperative chemoradiotherapy of esophageal carcinoma. Clinicopathologic correlations. Cancer. 1994;73:2680–2686. 8194005 10.1002/1097-0142(19940601)73:11<2680::aid-cncr2820731105>3.0.co;2-c

[30] Sobin L Wittekind C. International Union Against Cancer (UICC). TNM Classification of Malignant Tumours. 6th edition, Wiley; 2002.

[31] Sobin LH Fleming ID. TNM Classification of Malignant Tumors, fifth edition (1997). Union Internationale Contre le Cancer and the American Joint Committee on Cancer. Cancer. 1997;80:1803–1804. 9351551 10.1002/(sici)1097-0142(19971101)80:9<1803::aid-cncr16>3.0.co;2-9

[32] Frolov YS Maling DH. The accuracy of area measurement by point counting techniques. Cartogr J. 1969;6:21–35.

[33] Team RC. R: A Language and Environment for Statistical Computing. Vienna, Austria: R Foundation for Statistical Computing; 2017.

[34] Gauthier J Wu QV Gooley TA. Cubic splines to model relationships between continuous variables and outcomes: a guide for clinicians. Bone Marrow Transplant. 2020;55:675–680. 31576022 10.1038/s41409-019-0679-x

[35] Harrell FE Jr. Hmisc: Harrell Miscellaneous. R package, Version: 4.4-2; 2020.

[36] Okada K Sadahiro S Miyakita H. Relation between the size of nonmetastatic lymph nodes and outcomes in patients with stage III colorectal cancer. J Clin Oncol. 2018;36(Suppl 4):822.

[37] Markl B Schaller T Kokot Y. Lymph node size as a simple prognostic factor in node negative colon cancer and an alternative thesis to stage migration. Am J Surg. 2016;212:775–780. 26307422 10.1016/j.amjsurg.2015.05.026

[38] Johnson PM Porter GA Ricciardi R. Increasing negative lymph node count is independently associated with improved long-term survival in stage IIIB and IIIC colon cancer. J Clin Oncol. 2006;24:3570–3575. 16877723 10.1200/JCO.2006.06.8866

[39] Matsuno S Kobari M Hisano H. Prognosis of the pancreatic cancer in terms of the regional lymph node reaction. Tohoku J Exp Med. 1985;145:291–302. 4002218 10.1620/tjem.145.291

[40] Saldanha P. Morphological assessment of lymph nodes draining carcinoma. MGM J Med Sci. 2016;3:190–197.

[41] Chang KL Arber DA Weiss LM. Weidner N Cote RJ Suster S Weiss LM Lymph nodes [chapter 41] Modern Surgical Pathology. 2nd edition, Philadelphia: W.B. Saunders; 2009:1431–1511.

[42] Bajénoff M Breart B Huang AYC. Natural killer cell behavior in lymph nodes revealed by static and real-time imaging. J Exp Med. 2006;203:619–631. 16505138 10.1084/jem.20051474 PMC2118232

[43] Grigoriadis A Gazinska P Pai T. Histological scoring of immune and stromal features in breast and axillary lymph nodes is prognostic for distant metastasis in lymph node-positive breast cancers. J Pathol Clin Res. 2018;4:39–54. 29416876 10.1002/cjp2.87 PMC5783956

[44] Johnson SC Frattolin J Edgar LT. Modelling the effects of lymph node swelling on T-cell response. bioRxiv. 2020. 2020.06.19.161232.

[45] Chyou S Benahmed F Chen J. Coordinated regulation of lymph node vascular-stromal growth first by CD11c+ cells and then by T and B cells. J Immunol. 2011;187:5558–5567. 22031764 10.4049/jimmunol.1101724 PMC3221869

[46] He WZ Xie QK Hu WM. An increased number of negative lymph nodes is associated with a higher immune response and longer survival in colon cancer patients. Cancer Manag Res. 2018;10:1597–1604. 29950897 10.2147/CMAR.S160100 PMC6014727

[47] Okada K Sadahiro S Suzuki T. Effects of chemoradiotherapy on lymph nodes in patients with rectal adenocarcinoma: evaluation of numbers and sizes of retrieved lymph nodes inside and outside the radiation field. Anticancer Res. 2014;34:4195–4200. 25075046

[48] Schroder W Baldus SE Monig SP. Lymph node staging of esophageal squamous cell carcinoma in patients with and without neoadjuvant radiochemotherapy: histomorphologic analysis. World J Surg. 2002;26:584–587. 12098049 10.1007/s00268-001-0271-5

