# Supplementary material for: Prognostic Significance of Negative Lymph Node Long Axis in Esophageal Cancer: Results From the Randomized Controlled UK MRC OE02 Trial
Source: Ann Surg. 2023 Jan 10;277(2):e320–31. doi: 10.1097/SLA.0000000000005214 (PMC9831043; doi:10.1097/SLA.0000000000005214)
Supplement: Supplementary file 1 [file sla-277-e320-s001.docx]

*Supplemental Digital Content to:*

Maximilian Kloft*, Jessica E. Ruisch*, Gayatri Raghuram, Jake Emmerson, Matthew Nankivell, David Cunningham, William H. Allum, Ruth E. Langley, Heike I. Grabsch: Prognostic significance of negative lymph node long axis in esophageal cancer - results from the randomized controlled UK MRC OE02 trial

Table of Content

| **Table S-1.** REMARK checklist | 2 |
| --- | --- |
| **Table S-2.** REMARK profile | 3 |
| **Table S-3.** Overview of used cut-off points. | 3 |
| **Table S-4.** Comparison of our current cohort with the whole OE02 trial cohort of patients who had surgery. | 4 |
| **Table S-5**. Univariate overall survival comparison between the original cohort of patients who had a resection and our cohort. | 4 |
| **Table S-6** LNpos size and clinicopathological parameters. | 5 |
| **Figure S-1.** Examples of virtual slides of Hematoxylin-Eosin stained histopathological sections of lymph nodes. | 6 |
| **Figure S-2.** Example of point counting method | 7 |
| **Figure S-3.** Different microarchitectural features assessed by the point counting method. | 8 |
| **Figure S-4.** 5-year overall survival in the original cohort of patients who had a resection versus our cohort. | 9 |
| **Figure S-5**. 5-year overall survival of our patient cohort in the OE02 trial stratified by radiologic cut-off (10mm) size of the negative lymph node (LNneg). | 10 |

**Table S-1.** REMARK checklist

| **Item to be reported** | | **Page no.** |
| --- | --- | --- |
| **INTRODUCTION** | |  |
| 1 | State the marker examined, the study objectives, and any pre-specified hypotheses. | 3-4 |
| **MATERIALS AND METHODS** | |  |
| *Patients* | |  |
| 2 | Describe the characteristics (e.g., disease stage or co-morbidities) of the study patients, including their source and inclusion and exclusion criteria. | 5-6 |
| 3 | Describe treatments received and how chosen (e.g., randomized or rule-based). | 5 |
| *Specimen characteristics* | |  |
| 4 | Describe type of biological material used (including control samples) and methods of preservation and storage. | 5 |
| *Assay methods* | |  |
| 5 | Specify the assay method used and provide (or reference) a detailed protocol, including specific reagents or kits used, quality control procedures, reproducibility assessments, quantitation methods, and scoring and reporting protocols. Specify whether and how assays were performed blinded to the study endpoint. | n.a. |
| *Study design* | |  |
| 6 | State the method of case selection, including whether prospective or retrospective and whether stratification or matching (e.g., by stage of disease or age) was used. Specify the time period from which cases were taken, the end of the follow-up period, and the median follow-up time. | 5 |
| 7 | Precisely define all clinical endpoints examined. | 7 |
| 8 | List all candidate variables initially examined or considered for inclusion in models. | 6 |
| 9 | Give rationale for sample size; if the study was designed to detect a specified effect size, give the target power and effect size. *(all available samples were analyzed)* | 6 |
| *Statistical analysis methods* | |  |
| 10 | Specify all statistical methods, including details of any variable selection procedures and other model-building issues, how model assumptions were verified, and how missing data were handled. | 6-7 |
| 11 | Clarify how marker values were handled in the analyses; if relevant, describe methods used for cutpoint determination. | 6-7 |
| **RESULTS** | |  |
| *Data* | |  |
| 12 | Describe the flow of patients through the study, including the number of patients included in each stage of the analysis (a diagram may be helpful) and reasons for dropout. Specifically, both overall and for each subgroup extensively examined report the numbers of patients and the number of events. | 8; suppl. p. 3 |
| 13 | Report distributions of basic demographic characteristics (at least age and sex), standard (disease-specific) prognostic variables, and tumor marker, including numbers of missing values. | 8 |
| *Analysis and presentation* | |  |
| 14 | Show the relation of the marker to standard prognostic variables. | 8 |
| 15 | Present univariable analyses showing the relation between the marker and outcome, with the estimated effect (e.g., hazard ratio and survival probability). Preferably provide similar analyses for all other variables being analyzed. For the effect of a tumor marker on a time-to-event outcome, a Kaplan-Meier plot is recommended. | 9 |
| 16 | For key multivariable analyses, report estimated effects (e.g., hazard ratio) with confidence intervals for the marker and, at least for the final model, all other variables in the model. | 9-10 |
| 17 | Among reported results, provide estimated effects with confidence intervals from an analysis in which the marker and standard prognostic variables are included, regardless of their statistical significance. | 9-10 |
| 18 | If done, report results of further investigations, such as checking assumptions, sensitivity analyses, and internal validation. | n.a. |
| **DISCUSSION** | |  |
| 19 | Interpret the results in the context of the pre-specified hypotheses and other relevant studies; include a discussion of limitations of the study. | 11-12 |
| 20 | Discuss implications for future research and clinical value. | 13 |

**Table S-2**. REMARK profile

| Study marker | Remarks | | | |
| --- | --- | --- | --- | --- |
| Marker: | LNneg=Negative lymph node size (continuous) | | | |
| Further variables | Age at diagnosis^,^ sex, T status, N status, histology, location of primary tumor, grade of differentiation, lymphatic invasion, blood vessel invasion, resection margin status, tumor regression grade primary tumor | | | |
| Patients | N | Remarks | | |
| Assessed for eligibility | 802 | Patients recruited to the OE02 trial | | |
| Excluded | 435 | Surgery not performed (n=60), slides not received (n=430), no survival data available (n=3) | | |
| Included | 302 | Patients with LNneg measured and survival data available | | |
| With outcome events | 229 | Overall survival: death from any cause | | |
| Statistical analyses of survival outcomes | | | | |
| Analysis | Patients | Events | Variables considered | Results |
| A1: Univariate survival analysis | 302 | 226 (76 censored) | Age at diagnosis, treatment, location primary tumor, histology primary tumor, (y)pT, (y)pN, grade of differentiation, lymphatic invasion, blood vessel invasion, resection margin status, tumor regression grade of primary tumor  LNneg size (≥7.41mm versus LNneg <7.41mm) | Table 2  Figure 3 |
| A2: Restricted cubic spline approach | 302 | 226 (76 censored) | Hazard ratio in relationship to LNneg size | Figure 2 |
| A3: Multivariate survival analysis | 300 | 225 (75 censored, 2 missing variables) | T status, N status, LNneg size spline level, treatment, interaction LNneg*treatment | Table 2 |
| A4: Exploratory univariate analysis | 302 | 226 (76 censored) | Combined groups of N status and LNneg size (N0-small LNneg, N0-large LNneg, N1-small LNneg, N1-large LNneg) | Figure 4. |

**Table S-3**. Overview of used cut-off points.

| Patient group | Cut-off point | Analysis |
| --- | --- | --- |
| All patient in OE02 cohort (n=302) | 7.41 mm | Univariate survival analysis |
| N0 patients (n=93) | 8.13 mm | Analysis of microarchitecture of N0 patients |

**Table S-4**. Comparison of our current cohort with the whole OE02 trial cohort of patients who had surgery.

| Characteristics |  | Our cohort | Whole surgery cohort | | p-value | |  |
| --- | --- | --- | --- | --- | --- | --- | --- |
| Sex (%) | Male | 279 (76.0) | 324 (74.5) | | 0.62 | |  |
|  | Female | 88 (24.0) | 111 (25.5) | |  |  |  |
| Age at diagnosis (median) | | 62.57 | | 63.01 | | 0.46 | |

**Table S-5**. Univariate overall survival comparison between our current cohort and the whole OE02 trial cohort of patients.

|  | **Hazard ratio** | **95.0% Confidence interval** | | **p-value** |
| --- | --- | --- | --- | --- |
|  |  | Lower | Upper |  |
| Our cohort (n=367) vs whole OE02 trial cohort of patients who had surgery (n=381) | 0.92 | 0.78 | 1.09 | 0.33 |

|  |  |  |  |  |  |  |  |  |  |  |  |  |  |  |  |
| --- | --- | --- | --- | --- | --- | --- | --- | --- | --- | --- | --- | --- | --- | --- | --- |
|  |  | All patients  with LNpos | | Chemo + surgery patients | | | | | Surgery alone patients | | | | | | |
| Characteristics |  |  |  | LNpos size < 8.7mm | | LNpos size ≥ 8.7mm | | p-value | LNpos size <10.97mm | | LNpos size ≥ 10.97mm | | | | p-value |
|  |  | n | % | n | % | n | % |  | n | % | n | % | | |  |
| Sex | Male | 196 | 83 | 42 | 24 | 49 | 89 | 0.08 | 54 | 86 | 51 | 81 | | | 0.48 |
|  | Female | 40 | 17 | 13 | 76 | 6 | 11 |  | 9 | 14 | 12 | 19 | | |  |
| Age at diagnosis (median) |  | 63.7 | | 61.6 | | 63.8 | | 0.75 | 63.9 | | 64.4 | | | | 0.18 |
| Location of primary tumor | Lower | 166 | 70 | 42 | 76 | 38 | 69 | 0.3 | 41 | 65 | 45 | 71 | | | 0.53 |
|  | Middle | 39 | 17 | 10 | 18 | 9 | 16 |  | 12 | 19 | 8 | 13 | | |  |
|  | Upper | 31 | 13 | 3 | 6 | 8 | 15 |  | 10 | 16 | 10 | 16 | | |  |
| Histology of primary  tumor | AC | 189 | 80 | 44 | 80 | 44 | 80 | 0.67 | 54 | 86 | 47 | 75 | | | 0.06 |
|  | SCC | 41 | 17 | 11 | 20 | 10 | 19 |  | 6 | 10 | 14 | 22 | | |  |
|  | Other | 6 | 3 | 0 | 0 | 1 | 1 |  | 3 | 4 | 2 | 3 | | |  |
| (y)pT | T1 | 9 | 4 | 3 | 5 | 2 | 4 | 0.75 | 1 | 2 | 3 | 5 | | | 0.67 |
|  | T2 | 14 | 6 | 4 | 7 | 2 | 4 |  | 5 | 8 | 3 | 5 | | |  |
|  | T3 | 207 | 88 | 46 | 84 | 51 | 92 |  | 56 | 89 | 54 | 85 | | |  |
|  | T4 | 6 | 2 | 2 | 4 | 0 | 0 |  | 1 | 2 | 3 | 5 | | |  |
| (y)pN | N0 | 0 | 0 | 0 | 0 | 0 | 0 | 1.0 | 0 | 0 | 0 | 0 | | | 1.0 |
|  | N1 | 236 | 100 | 56 | 100 | 55 | 100 |  | 63 | 100 | 63 | 100 | | |  |
| Grade of differentiation | Moderate/Well | 110 | 31 | 29 | 53 | 25 | 45 | 0.53 | 34 | 54 | 34 | 54 | | | 0.94 |
|  | Poor | 122 | 52 | 24 | 44 | 29 | 53 |  | 29 | 46 | 28 | 44 | | |  |
|  | Unknown | 4 | 17 | 2 | 3 | 1 | 2 |  | 0 | 0 | 1 | 2 | | |  |
| Lymphatic invasion | Negative | 111 | 51 | 33 | 64 | 30 | 60 | 0.5 | 24 | 41 | 24 | 41 | | | 0.79 |
|  | Positive | 108 | 49 | 19 | 36 | 20 | 40 |  | 34 | 59 | 35 | 59 | | |  |
| Blood vessel invasion | Negative | 171 | 80 | 49 | 94 | 43 | 84 | 0.11 | 40 | 69 | 39 | 75 | | | 0.48 |
|  | Positive | 42 | 20 | 3 | 6 | 8 | 16 |  | 18 | 31 | 13 | 25 | | |  |
| Resection margin status | Negative | 128 | 54 | 27 | 49 | 32 | 58 | 0.17 | 35 | 56 | 34 | 54 | | | 0.5 |
|  | Positive | 83 | 35 | 24 | 44 | 16 | 29 |  | 19 | 30 | 24 | 38 | | |  |
|  | Unknown | 25 | 11 | 4 | 7 | 7 | 13 |  | 9 | 14 | 5 | 8 | | |  |
| Tumor regression grade  primary tumor | TRG 1-3 | 17 | 15 | 8 | 14 | 9 | 16 | 0.82 |  |  |  |  |  |  |  |
|  | TRG 4-5 | 92 | 84 | 46 | 84 | 46 | 84 |  |  |  |  |  |  |  |  |
|  | Unknown | 1 | 1 | 1 | 2 | 0 | 0 |  |  |  |  |  |  |  |  |

**Table S-6**. Clinicopathological characteristics for all patients with positive lymph nodes (LNpos), and for CS and S patients stratified by median LNpos size.

AC: Adenocarcinoma SCC: Squamous cell carcinoma *T0: No residual tumor in the specimen

Location primary tumor: refers to lower, middle and upper thirds of the esophagus

**Figure S-1.** Examples of virtual slides of Hematoxylin-Eosin stained histopathological sections of lymph nodes.

(A) Two tumor-free lymphoid tissues close to each other. The distance between the two lymphoid tissues is < 1mm (see ‘z’ line) and they are surrounded by one piece of fatty tissue, therefore the 2 lymphoid tissues were defined as ‘one’ LN. Line ‘x’ represents the largest diameter of the lymph node. Magnification: x1.5.

(B) One positive lymph node with a clearly visible capsule surrounding the lymphoid tissue. Line ‘x’ represents the largest diameter of the lymph node. Magnification: x0.6.


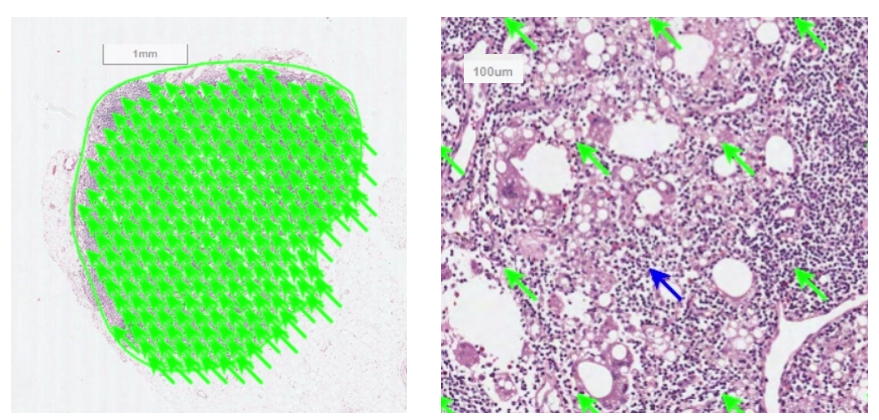


A

B

**Figure S-2.** Illustration of the point counting method using random systematic sampling.

1. Two hundred and fifty measurement points (=arrows) were automatically distributed within the annotation of a LNneg. Magnification: x0.5.
2. The tissue at the tip of the arrow was scored according to the five microarchitectural features. Magnification: x5.


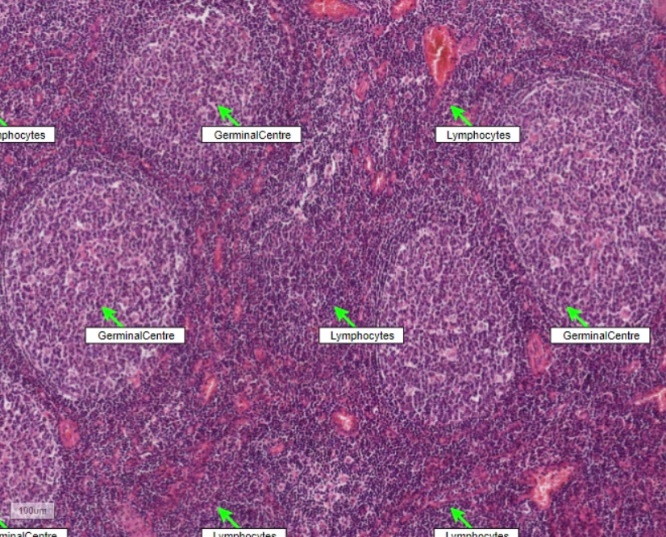

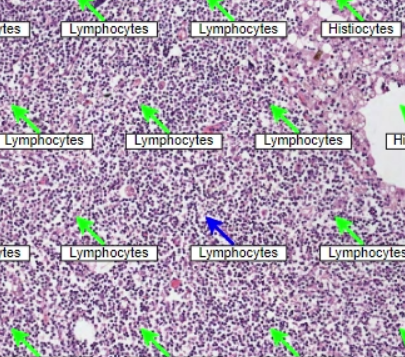


A

B


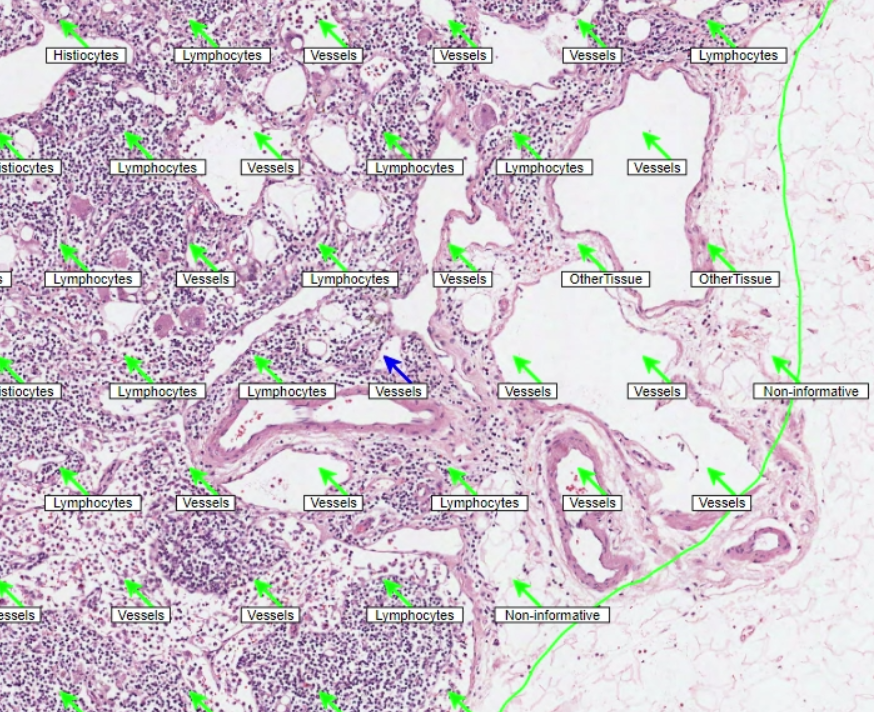

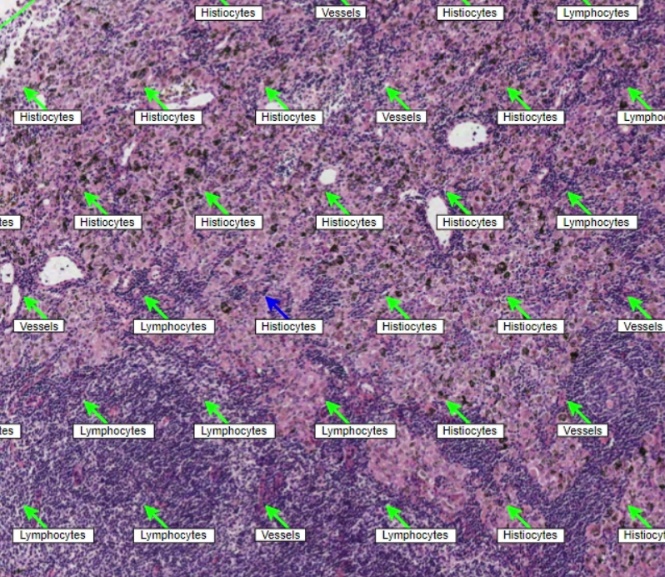


C

D


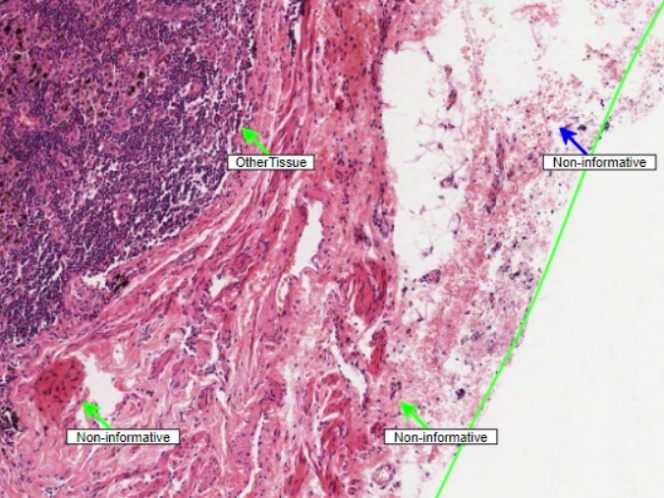

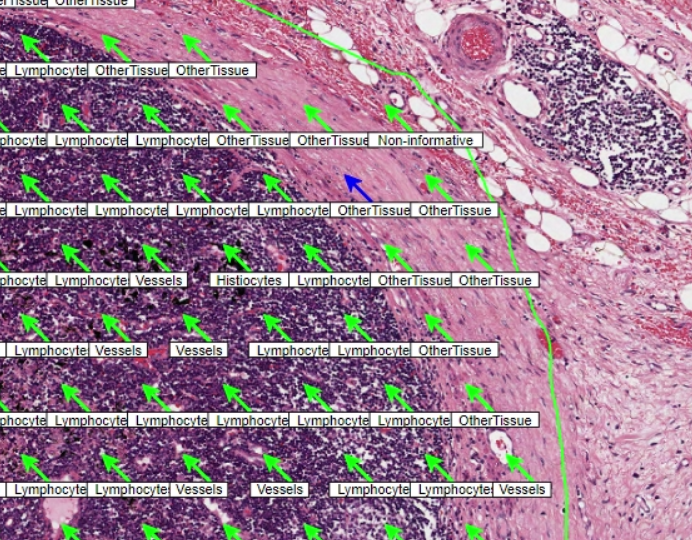


F

E

**Figure S-3.** Different microarchitectural features assessed by the point counting method.

A) Lymphocytes located outside germinal centers. B) Germinal centers. C) Histiocytes (tissue macrophages). D) Vessels. E) Other tissue (connective tissue of LN capsule). F) non-informative (tissue outside of LN capsule).


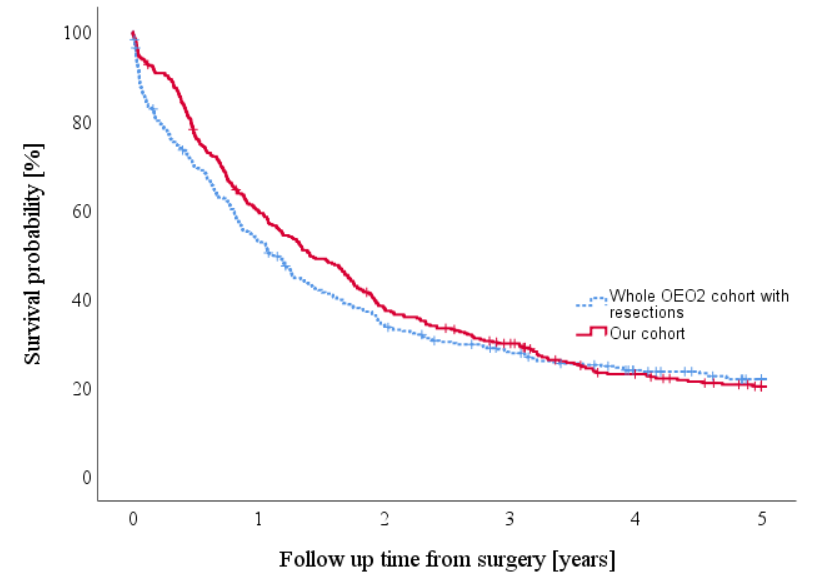


Survival probability [%]

| Number of patients at risk | |  |  |  |  |  |
| --- | --- | --- | --- | --- | --- | --- |
| Whole cohort | 381 | 198 | 126 | 97 | 74 | 59 |
| Our cohort | 367 | 214 | 134 | 101 | 69 | 52 |

**Figure S-4.** 5-year overall survival (OS) of our cohort versus the whole OE02 cohort of patients who had a resection. The Kaplan Meier plot shows there is no difference in OS esophageal cancer patients in our cohort versus those patients in the whole cohort who had a resection. HR:0.92, 95% CI: 0.78-1.09; p=0.33. 5-year OS: 20% vs. 21.8%.


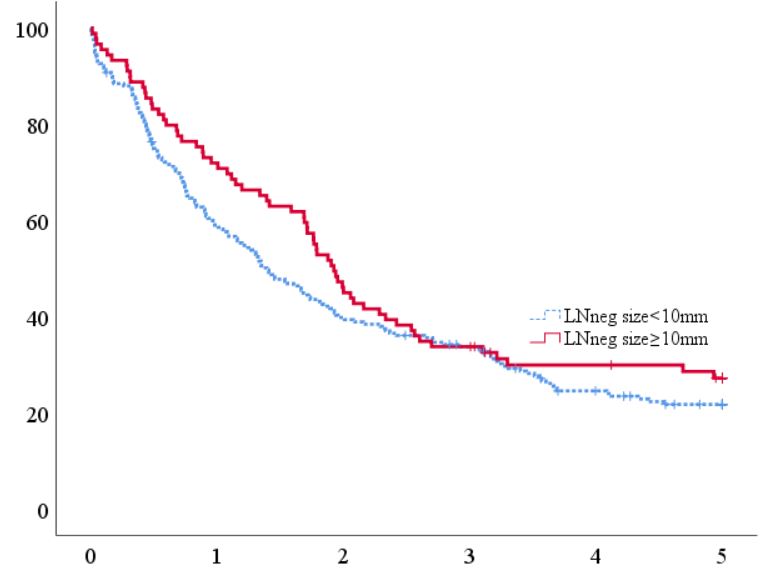


Follow up time from surgery [years]

Survival probability [%]

| Number of patients at risk | |  |  |  |  |  |
| --- | --- | --- | --- | --- | --- | --- |
| LNneg≥10mm | 89 | 63 | 39 | 29 | 22 | 18 |
| LNneg<10mm | 216 | 125 | 84 | 68 | 44 | 33 |

**Figure S-5.** 5-year overall survival (OS) stratified by radiology cut-off (10mm) of size of the negative lymph node (LNneg). The Kaplan Meier plot shows that the survival of esophageal cancer patients with LNneg size ≥10mm does not differ significantly from those with LNneg size <10mm. HR: 1.21, 95%, CI: 0.91-1.62; p=0.19, 5-year OS: 27.2% vs 21.7%.
